# Trends in lipid profiles in patients with psoriasis: a population-based analysis

**DOI:** 10.1186/1471-5945-12-20

**Published:** 2012-10-30

**Authors:** Bharath Manu Akkara Veetil, Eric L Matteson, Hilal Maradit-Kremers, Marian T McEvoy, Cynthia S Crowson

**Affiliations:** 1 Division of Rheumatology, Mayo Clinic, Mayo Clinic College of Medicine, Rochester, Minnesota, USA; 2 Department of Health Sciences Research, Mayo Clinic, Mayo Clinic College of Medicine, Rochester, Minnesota, USA; 3 Department of Dermatology, Mayo Clinic, Mayo Clinic College of Medicine, Rochester, Minnesota, USA

**Keywords:** Psoriasis, Lipids, Epidemiology

## Abstract

**Background:**

Psoriasis is associated with an atherogenic lipid profile but longitudinal changes in lipids around disease onset are unknown. The purpose of our study is to examine the effect of psoriasis onset on serum lipid profiles.

**Methods:**

We compared changes in lipid profiles in a population based incident cohort of 689 patients with psoriasis and 717 non-psoriasis subjects. All lipid measures performed 5 years before and after psoriasis incidence/index date were abstracted. Random-effects models adjusting for age, sex and calendar year were used to examine trends in lipid profiles.

**Results:**

There were significant declines in total cholesterol (TC) and low-density lipoprotein (LDL) levels during the 5 years before and after psoriasis incidence/index date in both the psoriasis and the non-psoriasis cohorts, with a greater decrease noted in the TC levels (p=0.022) and LDL (p=0.054) in the non-psoriasis cohort. High-density lipoprotein (HDL) levels increased significantly both before and after psoriasis incidence date in the psoriasis cohort. Triglyceride (TG) levels were significantly higher (p<0.001), and HDL levels significantly lower (p=0.013) in patients with psoriasis compared to non-psoriasis subjects. There were no differences in prescriptions for lipid lowering drugs between the two cohorts.

**Conclusions:**

Patients with psoriasis had a significant decrease in TC and LDL levels during the 5 years before psoriasis incidence. Higher mean TG and lower mean HDL levels were noted in the 5 years before psoriasis incidence. These changes are unlikely to be caused by lipid lowering treatment alone and require further exploration.

## Background

Psoriasis is becoming understood as a systemic and inflammatory disease with increased associated comorbidities, including risk for cardiovascular (CV) disease [1,2]. Among the comorbidities which predispose patients with psoriasis to increased risk of CV disease are psoriatic arthritis and sleep disorders [3], as well as other traditional risk factors for CV disease, including atherogenic lipid profiles [4,5].

Little is known about the impact of psoriasis on lipids in patients with new onset psoriasis. A number of studies have demonstrated a pro-atherogenic lipid profile in psoriasis, but not all [5-9]. There is now growing evidence that psoriasis is associated with enhanced atherosclerosis and unfavorable lipid profiles, but some of the conflicting results examining this relationship may be at least in part influenced by the effects of inflammation related to psoriasis, as well as its treatment [6].

To address the relationship between psoriasis and the profile of potentially atherogenic lipids, we performed a longitudinal study of changes in lipid profile during the period surrounding psoriasis incidence in a population-based cohort of patients with psoriasis and a comparison cohort of non-psoriasis subjects. The aim of this study was to determine the effect of psoriasis onset on serum lipid profiles by comparing lipid profiles in patients with psoriasis and non-psoriasis subjects during the 5 years before and 5 years after psoriasis incidence/index date.

## Methods

This retrospective longitudinal cohort study was performed using the population-based resources of the Rochester Epidemiology Project (REP) medical records linkage system. This records linkage system allows ready access to the complete medical records from all health care providers from the Mayo Clinic and its affiliated hospitals, the Olmsted Medical Center, the Olmsted Community Hospital, local nursing homes, and the few private practitioners. The potential of this data system for population-based studies has been described elsewhere [10-12]. This system ensures virtually complete ascertainment of all clinically recognized cases of psoriasis among the residents of Olmsted County, MN.

The study population comprised a retrospectively identified incidence cohort of Olmsted County, MN residents ≥35 years of age who were first diagnosed with psoriasis between January 1, 1988 and January 1, 2008, and have at least one lipid measure during the time period from 5 years before to 5 years after psoriasis incidence date. The complete medical records of all subjects were reviewed, and psoriasis was validated by either a confirmatory diagnosis in the medical record by a dermatologist, or a physician’s description of the lesions in the medical record or a skin biopsy, whenever available. For the majority of psoriasis subjects (551/689 = 80%), data was also collected on start and stop dates of all courses of systemic drug therapy, including methotrexate (MTX), oral retinoids, azathioprine, cyclosporine, hydroxychloroquine, sulfasalazine, leflunomide, and biologics (i.e., etanercept, infliximab, adalimumab, golimumab, efalizumab, alefacept).

In case of a doubtful diagnosis, the medical record was reviewed by the dermatologist co-investigator. Incidence date was defined as the physician diagnosis date. Subjects with prevalent psoriasis, subjects with missing medical records, and those who denied research authorization were excluded.

For each patient with psoriasis, a non-psoriasis subject of similar age, sex, calendar year and length of medical history prior to index date was randomly selected from the same population. Each non-psoriasis subject was assigned an index date corresponding to the psoriasis incidence date of the corresponding patient with psoriasis.

All lipid measures performed for clinical indications from 5 years prior to psoriasis incidence/index date to last follow-up were abstracted. These included total cholesterol (TC), high-density lipoprotein (HDL), low-density lipoprotein (LDL) and triglycerides [TG]). In accordance with Adult Treatment Panel III (ATPIII) guidelines [13], abnormal lipid levels were defined as TC ≥240 mg/dL, LDL ≥160 mg/dL, TG ≥200 mg/dL or HDL <40 mg/dL. Data on prescription of lipid-lowering medications (i.e., statins and other lipid-lowering drugs) and body mass index were also collected. Obesity was defined as body mass index ≥ 30 kg/m2. The study protocol was approved by the Institutional Review Boards from Mayo Clinic and Olmsted Medical Center.

### Statistical methods

Descriptive statistics (means, percentages, etc.) were used to summarize the lipid measures. Demographics of patients with psoriasis and non-psoriasis subjects, as well as subjects with and without lipid measures, were compared using Chi-square tests and t-tests. Random effects models adjusting for age, sex, and calendar year of psoriasis incidence/index date were used to analyze the trends in lipid profiles during the time period from 5 years before to 5 years after psoriasis incidence/index date. These models accounted for multiple measurements per subject using random effects to fit individual intercepts and slopes for each subject. Generalized additive models with smoothing splines were used to illustrate the trends in lipid levels over time.

## Results

A total of 963 Olmsted County, MN residents aged ≥35 years were diagnosed with psoriasis between 1/1/1988 and 1/1/2008. Of these, 689 patients had at least one lipid measure during the time period from 5 years before to 5 years after psoriasis incidence date and thus were included in the study (Table 1). The total number of lipid measurements in patients with psoriasis was 3,561 (median 3 measurements per patient). Patients with psoriasis without lipid measures during the time period of interest (n=274) were younger (mean age 51.9 years, p<0.001) and were more likely to be male (59.5% vs. 46.2%) than patients with psoriasis in whom lipid measures were obtained. Within the same time period, lipid measurements were also available for 717 non-psoriasis subjects with a total of 3,678 lipid measurements (median 3 measurements per subject). The spread of measurements were similar for both groups (mean 9.38 years, standard deviation (SD) 1.36 years for psoriasis and mean 9.32 years, SD 1.45 years for non-psoriasis (p=0.68). Non-psoriasis subjects had similar age and sex characteristics compared to patients with psoriasis with lipid measures. In addition, the prevalence of lipid-lowering drug use was similar among psoriasis and non-psoriasis subjects (37% vs. 39%, respectively; p=0.33). Data on systemic medication used was available in 551 of the 689 psoriasis subjects. Of these, 49 (8.9% of the 551) used systemic medication during the study period.

**Table 1:** Characteristics of patients with psoriasis and non-psoriasis subjects

**Variable**	**Non-psoriasis (N=717)**	**Psoriasis (N=689)**
Age at psoriasis incidence/index date (years), mean (±Standard Deviation)	55.2 (± 13.2)	55.6 (± 13.3)
Male, No. (%)	353 (49)	318 (46)
Observation time (years)		
Before incidence/index date, mean (median)	4.73 (5)	4.71 (5)
After incidence/index date, mean (median)	4.65 (5)	4.61 (5)
Lab measurements		
Total number	3678	3561
Number per subject before incidence/index date, mean (median)	2.1 (1)	2.0 (1)
Number per subject after incidence/index date, mean (median)	3.0 (2)	3.2 (2)
Use of statins and/or lipid lowering therapy, No. (%)	262 (37)	269 (39)
Obesity*	301 (46)	271 (39)

*Body mass index at psoriasis incidence or non-psoriasis index date was available in 660 psoriasis and 693 non-psoriasis subjects.

Demographic characteristics and length of follow-up were similar in both psoriasis and non-psoriasis subjects (Table 1). The median follow-up was 5 years in each cohort as the follow-up was truncated at 5 years for these analyses. A full 5 years of follow-up after psoriasis incidence/index date was available for 533 (77%) of patients with psoriasis and 563 (79%) of non-psoriasis subjects. In addition, 628 (91%) of psoriasis and 650 (91%) of non-psoriasis subjects had 5 years of available information prior to psoriasis incidence/index date. The mean time prior to psoriasis incidence/index date was 4.7 years in both groups with a median of 5 years.

Table 2 shows the mean difference in lipid levels between the psoriasis and non-psoriasis cohorts adjusted for age, and calendar year. Overall, TG levels were significantly higher in psoriasis compared to non-psoriasis subjects (on average 16.8 mg/dL; p<0.001) and HDL levels were significantly lower in patients with psoriasis compared to non-psoriasis subjects (−1.9 mg/dL; p=0.013). Subgroup analyses were performed for males and females, and non-obese and obese subjects, as well as statin users. Differences in TG levels between psoriasis and non-psoriasis subjects were more pronounced among males, obese subjects and statin users, and less pronounced among females, non-obese patients and those who did not use statins. No difference in mean lipid levels of TC or LDL between psoriasis and non-psoriasis subjects were noted overall or in any subgroups of patients.

**Table 2:** Mean differences in lipid levels in psoriasis patients compared to non-psoriasis subjects according to sex and statin use

**Lipid measure**	**Overall**	**Sex**		**Obesity**		**Statin use**	
		**Female**	**Male**	**Non-obese**	**Obese**	**Non-user**	**User**
Total Cholesterol (TC)	0.6	3.0	−1.8	0.1	0.4	0.4	3.4
Low-density Lipoprotein	−0.9	−1.3	−0.2	0.1	−2.3	−1.1	−0.7
High-density Lipoprotein (HDL)	−1.9*	2.2*	−6.1*	−1.7	−1.1	−2.1*	−1.3
Triglycerides	16.8*	8.6	25.2*	7.6	21.4*	17.7*	29.4*
TC: HDL ratio	0.2*	−0.1	0.5*	0.2*	0.1	0.2*	0.2

* p<0.05, indicates significant difference in mean lipid levels between psoriasis and non-psoriasis cohorts adjusted for age and calendar year. Values in table are mean differences in psoriasis minus non-psoriasis cohorts in mg/dL.

**Figure 1:**
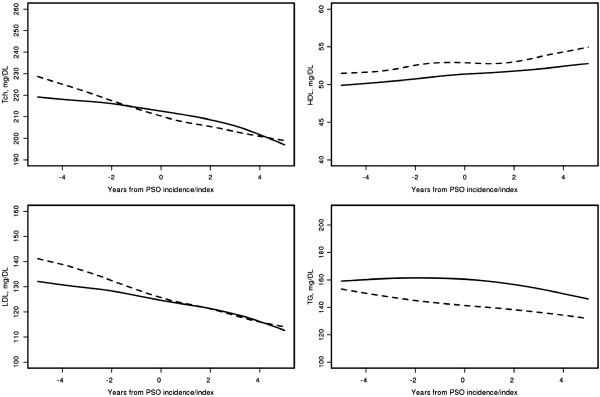
**Trends in lipid levels in psoriasis patients (solid lines) and non-psoriasis subjects (dashed lines) during the time period from 5 years before to 5 years after psoriasis incidence/index date adjusting for age, sex and calendar year of psoriasis diagnosis** .

Figure 1 and Table 3 show the trends in lipids during the 5 years before and 5 years after psoriasis incidence/index date in the psoriasis and non-psoriasis cohorts. There were significant declines in TC and LDL levels during the 5 years before and the 5 years after psoriasis incidence/index date in both the psoriasis and the non-psoriasis cohort. HDL levels increased significantly both before and after psoriasis incidence date in the psoriasis cohort. There was no significant change in HDL during the 5 years before index date in the non-psoriasis cohort. Subgroup analyses were performed for statin users. Statin users experienced significant declines in TC and LDL in both the psoriasis and non-psoriasis cohorts, as expected. However, there were no differences in the trends in lipids between the psoriasis and non-psoriasis groups among the subgroup of patients taking statins.

**Table 3:** Mean changes in lipid levels in psoriasis patients and non-psoriasis subjects from 5 years before to 5 years after psoriasis incidence/index date based on random effects models

**Lipid measure**	**Five-year change before the psoriasis incidence/index date**		**Five-year change after the psoriasis incidence/index date**	
	**Psoriasis**	**Non-psoriasis**	**Psoriasis**	**Non-psoriasis**
Total Cholesterol (TC)	−6.2 (−11.7, -0.7)*	−15.3 (−20.7, -9.9)	−15.9 (−20.3, -11.4)	−13.6 (−17.8, -9.5)
Low-density Lipoprotein	−8.3 (−13.0, -3.6)	−14.2 (−19.2, -9.2)	−13.2 (−17.1, -9.2)	−13.2 (−16.8, -9.5)
High-density Lipoprotein (HDL)	2.1 (0.8, 3.4)	1.2 (−0.1, 2.6)	1.4 (0.2, 2.5)	1.7 (0.6, 2.9)
Triglycerides	1.6 (−13.5, 16.6)	−7.0 (−18.0, 4.0)	−20.2 (−31.3, -9.0)	−15.8 (−24.5, -7.2)
TC: HDL ratio	−0.3 (−0.4, -0.1)	−0.4 (−0.6, -0.2)	−0.5 (−0.7, -0.4)	−0.5 (−0.6, -0.3)

* (p=0.024) indicates difference between the trends in lipid changes between psoriasis and non-psoriasis cohorts. Values in parentheses are 95% confidence intervals.

## Discussion

Both psoriasis and dyslipidemia are risk factors for cardiovascular disease in patients with psoriasis. To examine this relationship, we performed this retrospective study of lipid profiles during the period surrounding psoriasis incidence in a population-based cohort of patients with psoriasis and a comparison cohort of non-psoriasis subjects. There were significant declines in TC and LDL levels during the 5 years before and the 5 years after psoriasis incidence/index date in both the psoriasis and the non-psoriasis cohorts with a greater decrease noted in the TC levels and LDL in the non-psoriasis cohort. HDL levels increased significantly both before and after psoriasis incidence in the psoriasis cohort, but significant increases were seen only in the 5 years after index date for the non-psoriasis cohort. TG levels showed an increase in the 5 year period before psoriasis incidence in the psoriasis cohort and a decline prior to index date in the non-psoriasis cohort, but they did not achieve statistical significance. Lipid trends during the 5 years after incidence/index date were largely similar in both cohorts, with a decrease in TC, LDL and TG and an increase in HDL, with no significant differences between the trends in both groups.

There is conflicting information about how lipid profiles might be affected by psoriasis. An atherogenic lipid profile namely, higher cholesterol, LDL and TG levels were noted in psoriasis patients in some studies [5,7], with no significant difference between patients and controls noted in others [7-9]. It is also unknown whether the observed lipid changes are primary or secondary to the chronic inflammatory process or its treatment [6,14].

We sought to examine the potential relationship of psoriasis and dyslipidemia by examining lipid profiles in patients around the time of psoriasis onset. As far as we can ascertain, this is the first population-based study to describe longitudinal lipid trends in psoriasis and non-psoriasis populations both before and after psoriasis incidence/index date. The data from our study show that although there is indeed a downward trend in TC and LDL in both populations in the five years before psoriasis incidence/index date, the decline in TC and LDL levels was smaller in the psoriasis patients than in the non-psoriasis subjects. This atherogenic trend in the psoriasis subjects is further demonstrated by significantly increased TG levels and significantly lower HDL levels in psoriasis subjects compared to non-psoriasis subjects over the duration of the study.

There is an emerging consensus as to the role of the chronic inflammatory state in diseases like systemic lupus erythematosus and rheumatoid arthritis and the accompanying proinflammatory milieu in promoting development and progression of dyslipidemia and atherosclerosis. It is likely that psoriasis, a chronic immune mediated inflammatory skin disease, may predispose individuals to dyslipidemia [15]. This association is demonstrably stronger for severe psoriasis and psoriatic arthritis [5,15]. Psoriasis has also been shown to be an independent risk factor for cardiovascular mortality [16,17]. In addition, there appears to be a significant association between psoriasis and traditional risk factors for atherosclerosis and heart disease in the general population such as diabetes mellitus type II, coronary artery disease, peripheral vascular disease and hypertensive heart disease [13,15,16,18,19].

In our study, patients with psoriasis tended to have a smaller decline in the TC and LDL levels compared to non-psoriasis subjects in the 5 years before psoriasis incidence/index date, a finding which is concordant with some previous studies [5-7]. Direct comparisons with these findings cannot be made due to the differences in study design, time periods and populations involved. The downward trend in TC and LDL prior to psoriasis incidence is not without precedent and has also been demonstrated in other chronic inflammatory conditions such as rheumatoid arthritis [20]. The apparent paradox of downward trending total cholesterol levels and increased cardiovascular mortality risk in psoriasis may be explained by the altered cytokine milieu [16,19,21,22] and inflammation [23]. This is also consistent with the lowering of the plasma cholesterol concentrations seen in chronic inflammatory conditions [24,25].

The lowering of TC and LDL in the 5 years prior to psoriasis incidence was unlikely to have been caused by treatment for psoriasis. The broad downward trend in lipids in both the patients with psoriasis and the comparator subjects who did not have psoriasis could be partly explained by the increasing usage of lipid-lowering drugs in the general population during time period covered by the study [26]. A similar explanation might be suggested for the changes in HDL. However, the changes probably cannot be attributed solely to lipid lowering medications, as there was no difference in the number of subjects on lipid lowering medications in either cohort. An effect of onset of psoriasis contributing to the less marked decline in TC and LDL, with significantly higher TG and lower HDL, compared to non-psoriasis subjects cannot be excluded.

Strengths of this study include the longitudinal population based study design using the data from the Rochester Epidemiology Project with comprehensive data collection of the target population. We analyzed lipid trends before and after the incidence of psoriasis to ascertain longitudinal trends in these subjects. It is possible that patients with psoriasis had greater provider contact leading to greater number of lipid measurements and interventions. However, no differences in the number of lipid measures per person in patients with and without psoriasis were noted, and multiple measurements per person were accounted for during statistical analysis.

The population of Olmsted County, Minnesota is predominantly white, so that results may not be generalizable to non-white subjects. Except for the higher proportion of the population with higher educational levels, other sociodemographic characteristics of Olmsted County, Minnesota residents are similar to those of US white subjects. Further research is needed to assess the impact of traditional cardiovascular risk factors, comorbidities, psoriasis disease severity, and the choice of lipid-lowering therapy on the trends in lipids in patients with psoriasis. However, our subgroup analyses comparing patients with and without psoriasis among subgroups of patients with obesity and statin-users somewhat elucidate these issues. We were unable to perform an analysis of lipid trends by psoriasis disease severity score, as data on the severity and extent of skin involvement were not consistently available in this retrospective study. In addition, temporary changes in lipids due to acute illness are possible, and we were unable to exclude such lipid measurements from our analyses. However, acute illness is relatively rare and measurement of lipids are unlikely to occur as part of the clinical care during an acute illness; therefore, this issue should have little impact on or results.

## Conclusions

Patients with psoriasis had a significant decrease in TC and LDL levels during the 5 years before psoriasis incidence but the magnitude of the reduction was smaller than that seen in non-psoriasis subjects. Although there was no difference in the trends of TG and HDL levels between the two cohorts, mean TG levels were significantly higher in psoriasis subjects compared with non-psoriasis subjects. HDL levels were significantly lower in patients with psoriasis compared with non-psoriasis subjects. Lipid trends were otherwise similar in psoriasis and non-psoriasis cohorts during the 5 years after psoriasis incidence/index date. These changes are unlikely to be caused by lipid lowering treatment alone. The cause and implications of the apparent changes in lipid profile before psoriasis incidence require further exploration.

## Competing interests

Bharath Manu Akkara Veetil – None/None

Eric L Matteson – Grant from Pfizer/None

Hilal Maradit-Kremers – Grant from Amgen/None

Marian T McEvoy – None/None

Cynthia S Crowson – Grant from Pfizer, Amgen/None

## Authors’ contributions

BMAV participated in interpretation of the data and drafted the manuscript. ELM was involved in the conception of the study, participated in interpretation of the data and revision of the manuscript. HMK and MTM participated in acquisition of data, interpretation of the data, and revision of the manuscript. CSC was involved in the conception of the study, acquisition of data, statistical analysis and interpretation of the data and revision of the manuscript. All authors gave final approval of the version to be published.

## Pre-publication history

The pre-publication history for this paper can be accessed here:

http://www.biomedcentral.com/1471-5945/12/20/prepub
